# Polydnavirus Ank Proteins Bind NF-κB Homodimers and Inhibit Processing of Relish

**DOI:** 10.1371/journal.ppat.1002722

**Published:** 2012-05-24

**Authors:** Kavita Bitra, Richard J. Suderman, Michael R. Strand

**Affiliations:** Department of Entomology, University of Georgia, Athens, Georgia, United States of America; Stanford University, United States of America

## Abstract

Recent studies have greatly increased understanding of how the immune system of insects responds to infection, whereas much less is known about how pathogens subvert immune defenses. Key regulators of the insect immune system are Rel proteins that form Nuclear Factor-κB (NF-κB) transcription factors, and inhibitor κB (IκB) proteins that complex with and regulate NF-κBs. Major mortality agents of insects are parasitoid wasps that carry immunosuppressive polydnaviruses (PDVs). Most PDVs encode ank genes that share features with IκBs, while our own prior studies suggested that two ank family members from *Microplitis demolitor* bracovirus (MdBV) (Ank-H4 and Ank-N5) behave as IκB mimics. However, the binding affinities of these viral mimics for Rel proteins relative to endogenous IκBs remained unclear. Surface plasmon resonance (SPR) and co-immunoprecipitation assays showed that the IκB Cactus from *Drosophila* bound Dif and Dorsal homodimers more strongly than Relish homodimers. Ank-H4 and –N5 bound Dif, Dorsal and Relish homodimers with higher affinity than the IκB domain of Relish (Rel-49), and also bound Relish homodimers more strongly than Cactus. Ank-H4 and –N5 inhibited processing of compound Relish and reduced the expression of several antimicrobial peptide genes regulated by the Imd signaling pathway in *Drosophila* mbn2 cells. Studies conducted in the natural host *Pseudoplusia includens* suggested that parasitism by *M. demolitor* also activates NF-κB signaling and that MdBV inhibits this response. Overall, our data provide the first quantitative measures of insect and viral IκB binding affinities, while also showing that viral mimics disable Relish processing.

## Author Summary

Central to the study of host-pathogen interactions is understanding how the immune system of hosts responds to infection, and reciprocally how pathogens subvert host defenses. In the case of insects, understanding of how the immune system responds to infection greatly exceeds understanding of pathogen counterstrategies. Parasitoid wasps are key mortality agents of insects. Thousands of wasp species have also evolved a symbiotic relationship with large DNA viruses in the family Polydnaviridae whose primary function is to deliver immunosuppressive virulence genes to the insect hosts that wasps parasitize. The function of most PDV-encoded virulence genes, however, remains unknown. In this article, we investigated the function of two *ank* gene family members from *Microplitis demolitor* bracovirus (MdBV). Our results indicate that Ank-H4 and Ank-N5 function as mimics of IκB proteins, which regulate a family of transcription factors called NF-κBs that control many genes of the insect immune system. IκBs and NF-κBs also function as key regulators of the mammalian immune system. Our results thus suggest that viral Ank proteins subvert the immune system of host insects by targeting conserved signaling pathways used by a diversity of organisms.

## Introduction

The innate immune system defends insects against a diversity of potential pathogens [1]. As part of this system, the Toll and Imd pathways activate Nuclear Factor-κB (NF-κB) transcription factors, which regulate the expression of antimicrobial peptides (AMPs) and many other genes [2]–[6]. Both pathways have also been implicated in defending insects against both microbes (viruses, bacteria, fungi, protozoans) and multicellular parasites (nematodes, parasitoid wasps) [2], [7]–[13].

All NF-κBs are homo- or heterodimers of Rel proteins, which share a Rel homology domain (RHD) essential for dimerization and DNA binding [14]. In the absence of immune challenge, most NF-κBs form inactive complexes with Inhibitor κB (IκB) proteins that bind the RHD through an ankyrin repeat domain (ARD) [14]–[16]. In *Drosophila melanogaster*, activation of the Toll pathway by pathogen recognition signals causes NF-κBs comprised of Dif and/or Dorsal to dissociate from the IκB Cactus and translocate to the nucleus [17]–[19]. Activation of the Imd pathway in contrast induces caspase 8-mediated cleavage of the compound protein Relish (Rel-110), which results in its N-terminal, RHD-containing fragment (Rel-68) forming NF-κBs that translocate to the nucleus, and its C-terminal IκB fragment (Rel-49) remaining in the cytoplasm [20], [21].

Reciprocally, pathogens often evolve sophisticated counterstrategies for overcoming host immune defenses [22]–[28]. Among insects, thousands of parasitoid wasp species depend upon large DNA viruses in the family Polydnaviridae to parasitize hosts [28]. All parasitoid wasps lay their eggs into or on the body of another arthropod (the host), and their offspring develop by feeding on host tissues. Most polydnavirus (PDV)-carrying wasps parasitize larval stage Lepidoptera (moths and butterflies), with each wasp species carrying a genetically unique PDV and naturally parasitizing only one or a small number of host species [29]. PDVs persist in wasps and are transmitted to offspring as stably integrated proviruses [28]. Replication in contrast only occurs in the reproductive tract of females where virions accumulate to high densities. Wasps inject a quantity of these virions into hosts when laying eggs, which rapidly infect hemocytes, the fat body, and other tissues. Viral gene products thereafter prevent host immune defenses from killing the wasp's progeny, yet no viral replication occurs in hosts because the encapsidated form of the viral genome lacks essential genes required for virion formation [28], [30], [31]. PDVs are thus beneficial symbionts of wasps that function as replication-defective vectors for delivery of virulence genes to hosts.

*Microplitis demolitor* parasitizes the non-model lepidopteran *Pseudoplusia includens* and carries *M. demolitor* bracovirus (MdBV) whose 190 kb genome encodes 51 genes for proteins larger than 100 amino acids [32]. Most of these genes are expressed in *P. includens* hemocytes and fat body within 2 h of infection [33], and functional studies implicate several of these genes in disrupting encapsulation, phagocytosis, and melanization [34]–[38]. Some of these genes also belong to a multimember family called *ank* genes that share an IκB-like ARD but lack the phosphorylation and ubiquitination domains that regulate the dissociation and degradation of insect IκBs after immune challenge [32], [39]. Comparative genomic data indicate that Rel proteins and other components of the Toll and Imd pathways are conserved among insects including Lepidoptera. However, in the absence of any data on NF-κB/IκB binding interactions in *P. includens*, we previously used *Drosophila* Rel proteins to assess whether MdBV Ank proteins function as IκB mimics. Co-immunoprecipitation experiments indicated that two family members, Ank-H4 and Ank-N5, complex with Dif, Dorsal, and Relish. Gel shift assays further showed that these Ank proteins prevent NF-κBs containing Dif or Dorsal from binding to the κB site in the drosomysin promoter and also prevent NF-κBs containing processed Relish from binding to the κB site in the cecropinA1 promoter [39]. Taken together, these findings indicate that Ank-H4 and –N5 disrupt both Toll and Imd pathway signaling. However, these data provide no insight on the relative affinity of these Ank proteins for different Rel protein dimers in comparison to endogenous IκBs. They also provide no insight on whether Ank proteins disable Imd signaling by disabling Relish function before or after processing. Here, we show that Ank proteins compete with endogenous IκBs for binding to Relish, block processing of Rel-110, and reduce the expression of AMP genes regulated by the Imd pathway. Our results also reveal that *M. demolitor* induces the expression of AMP genes in *P. includens* that are likely regulated by NF-κB signaling, but MdBV inhibits this response.

## Results

### Expression and purification of recombinant IkBs and Rel proteins

**Figure 1:**
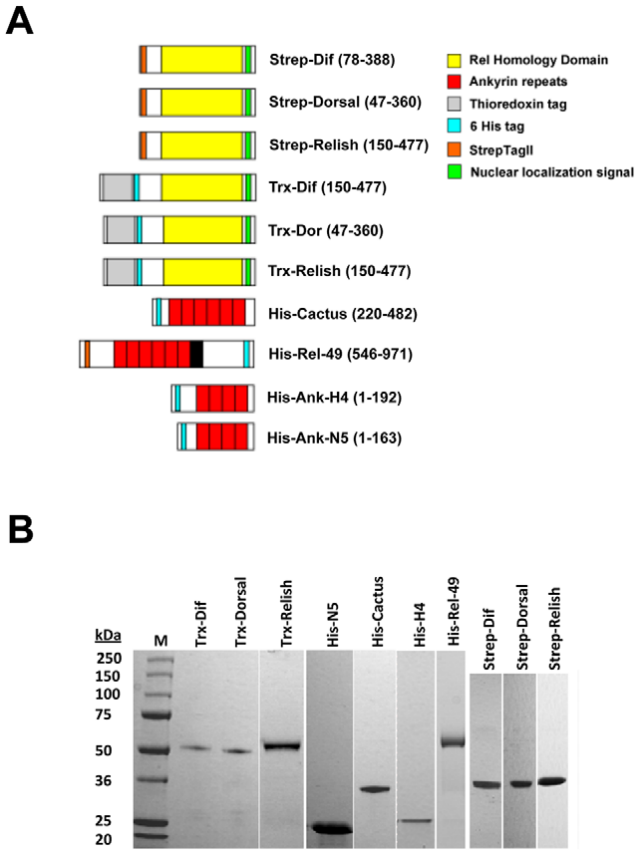
Recombinant Rel (Dif, Dorsal, Relish), IκB (Cactus, Rel-49) and viral Ank (Ank-H4, Ank-N5) constructs. (A) Domain structure of recombinant proteins. Each Rel protein contains the Rel Homology Domain and Nuclear localization signal plus either a Strep or Thioredoxin (Trx) epitope tag. Each IκB or Ank protein contains its Ankyrin Repeat Domain plus a 6× histidine (His) tag. Recombinant Rel-49 contained a Strep and His tag plus a PEST sequence (black) downstream of its ankyrin repeats. The numbers in parentheses next to each construct indicate the amino acid positions in the mature protein as indicated by their Genbank Accession numbers: Dif (AAA28465), Dorsal (AAA28479), Relish (AAF54333), Cactus (LD10168), Rel49 (same as Relish), Ank-H4 (AY875685) and Ank-N5 (AY875689). (B) SDS-PAGE analysis of each purified recombinant protein. Approximately 1 µg of each protein was loaded per lane. Molecular mass markers (M) labeled in kilodaltons (kDa) are indicated in the first lane to the left.

Rel proteins from mammals require the N-terminal RHD plus a downstream NLS for IκB binding [15], [16], [40]. In contrast, neither dimerization nor NF-κB/IκB binding requires any post-translational modifications or regions outside the RHD and NLS [16], [41]–[50]. We therefore used *E. coli* to express truncated forms of Dif, Dorsal, and Relish from *Drosophila* that contained the RHD, NLS and 20 additional C-terminal residues with a C-terminal StrepTagII tag (Figure 1A). These products were used in surface plasmon resonance (SPR) assays. We also produced truncated Rel proteins as N-terminal thioredoxin fusion constructs where the increased size allowed us to more easily distinguish them from IκBs in co-immunoprecipitation experiments (Figure 1A). Since only the ARD is required for IκB binding to NF-κBs [15], [16], we expressed a truncated form of Cactus that consisted of its ARD plus an N-terminal His tag (Figure 1A). In the absence of any information about binding of the IκB domain of Relish (Rel-49), we expressed a full-length version of Rel-49 with an N-terminal StrepTagII tag, and a C-terminal His tag (Figure 1A). Since MdBV Ank-H4 and –N5 consist of only an ARD [39], we expressed full-length versions of these proteins with N-terminal His tags (Figure 1A). Proteins were purified to greater than 90% purity as measured by loading at least 15 µg of protein on SDS-PAGE followed by Coomassie staining. Loading 1 µg of each recombinant protein on SDS-PAGE gels followed by Coomassie staining also confirmed that their size fully agreed with predicted masses (Figure 1B). The quaternary state of each purified recombinant protein was also analyzed by gel filtration, which as expected showed that each Rel protein formed homodimers as determined by comparison with molecular mass standards.

### *Drosophila* IκBs and MdBV Ank proteins bind homodimeric NF-κBs

Understanding of NF-κB/IκB binding interactions derives primarily from the study of mammalian Rel (p65, RelB, c-Rel, and the compound proteins p100, and p105) and IκB (IκBα, IκBβ, IκBε, Bcl-3, and C-terminal domains of p105 and p100) family members. This literature indicates that Rel proteins form different homo- and heterodimers and that IκB family members exhibit a gradient of binding preferences for different Rel complexes. For example, IκBα and IκBβ preferentially bind p50-p65 and p50-c-Rel heterodimers, IκBε binds homo and heterodimers containing p65, and Bcl-3 binds p50 and p52 homodimers [40], [45], [48], [51], [52]. Although the IκB domain of p105 binds its corresponding Rel domain (p50) after cleavage, it remains unclear whether binding occurs as part of a compound protein, after cleavage, or both [53]. Current understanding of NF-κB/IκB binding interactions in insects in contrast is both more limited and restricted to family members from *Drosophila*. Co-immunoprecipitation experiments and transgenic assays indicate that Dif, Dorsal, and Relish form all combinations of homo- and heterodimers [4], [54], [55]. Dif and Dorsal co-immunoprecipitate Cactus [18], [19], [56], but it remains unknown whether Cactus binds Rel protein dimers containing Relish. It also remains unknown whether unprocessed Relish (Rel-110) or its C-terminal IκB domain (Rel-49) bind any Rel protein dimer [57].

**Figure 2:**
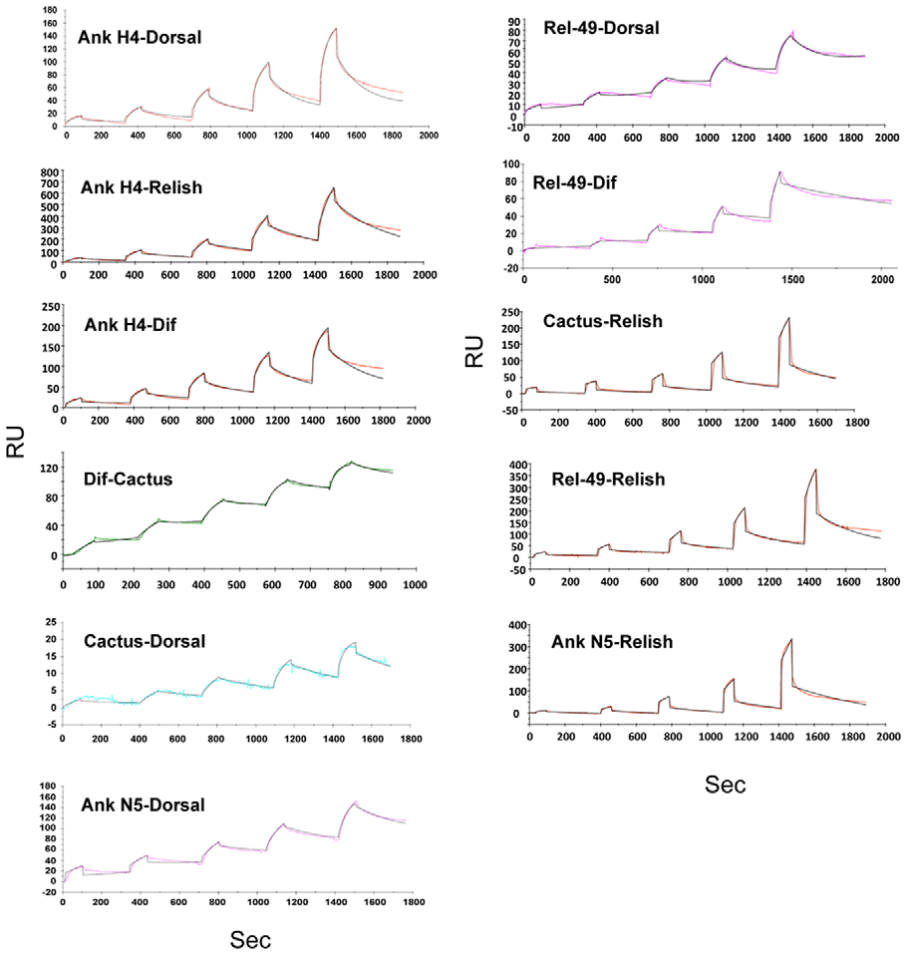
Kinetic titration sensorgrams of NF-κB/IκB interactions. Each recombinant Rel protein homodimer was injected sequentially over a surface with immobilized Cactus, Rel-49, Ank-H4, or Ank-N5 for 60 or 90 s at a flow rate of 30 µL per min, with a 120 s dissociation phase between injections. The concentration of NF-κB was doubled each injection. The sensorgram represents the response units (RU) from the blank-subtracted IκB coated surface (ordinate) with respect to time in seconds (abscisa). The dark lines represent the experimental data and the red lines represent the fit to a simple 1∶1 interaction model. See Table 1 for the association rate constants (ka), dissociation rate constants (kd), and equilibrium dissociation constants (Kd) in decreasing order of affinity.

Given the limited literature for insects, we conducted SPR assays that measured binding of Ank-H4, Ank-N5, Cactus, and Rel-49 to Dif, Dorsal and Relish homodimers. We also used the kinetic titration method to determine kinetic and thermodynamic constants and circumvent potential problems with non-specific binding and regeneration [58]. Recombinant IκB or Rel homodimers were immobilized on CM5 chips by amine coupling, followed by five sequential injections of doubling concentrations of a given Rel protein or IκB, which served as the analyte. We then generated sensograms by subtracting the response of a reference cell with no IκB from the response of the cell with the immobilized IκB. Our results indicated that each Ank and IκB bound the three Rel protein homodimers we assayed with the exception of Ank-N5, which did not bind Dif (Table 1, Figure 2). The strongest binding interaction we measured was between Cactus and Dif with a Kd of 110 nM, (Table 1). This value reflected a modest association rate (ka = 5.25×10^4^ M^−1^ s^−1^) and a very slow off rate (kd = 5.8×10^−3^ s^−1^). The affinity of Cactus for Dorsal (Kd = 195 nM) was slightly lower than for Dif and was much lower for Relish (Kd = 2.19 µM). Rel-49 modestly bound Dorsal (Kd = 783 nM) but weakly bound Relish (Kd = 2.75 µM). Compared to Cactus, Ank-H4 displayed a much higher binding affinity for Relish (Kd = 345 nM) and lower binding affinities for Dif (Kd = 581 nM) and Dorsal (Kd = 858 µM). With the exception of Dorsal, Ank-H4 also displayed higher binding affinities for each Rel homodimer than Ank-N5 (Table 1).

**Table 1:** SPR kinetic values for interactions between *Drosophila* IκB, viral Ank protein, and *Drosophila* NF-κB constructs.

Ligand	Analyte	ka (M^−1^ s^−1^)	kd (s^−1^)	Kd (M)	X^2^	Rmax
Dif	Cactus	5.25E+04	5.78E−03	1.10E−07	2.8	57
Cactus	Dorsal	9.89E+03	1.93E−03	1.95E−07	0.3	16
H4	Relish	1.94E+04	6.70E−03	3.45E−07	198.0	735
N5	Dorsal	7.23E+03	3.00E−03	4.16E−07	12.8	104
H4	Dif	6.62E+03	3.84E−03	5.81E−07	13.3	156
Rel-49	Dorsal	8.24E+03	6.45E−03	7.83E−07	3.6	43
H4	Dorsal	7.81E+03	6.70E−03	8.58E−07	10.5	124
Rel-49	Dif	5.24E+02	9.66E−04	1.84E−06	6.8	640
Cactus	Relish	1.10E+03	2.41E−03	2.19E−06	4.3	524
Rel-49	Relish	1.38E+03	3.78E−03	2.75E−06	12.9	467
N5	Relish	5.90E+01	1.79E−03	3.03E−05	86.0	6290
N5	Dif			No binding		

**Figure 3:**
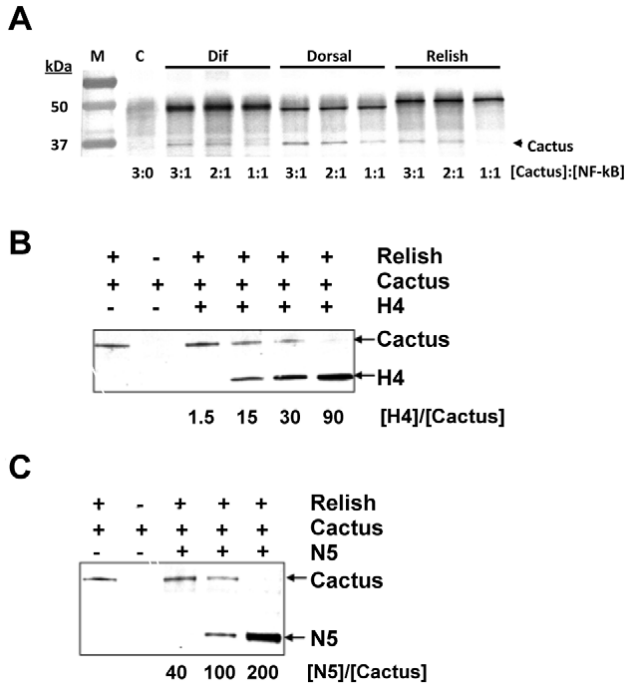
Recombinant Cactus binds recombinant Rel protein homodimers but binding to Relish is competed by Ank-H4 and Ank-N5. (A) Outcome of co-immunoprecipitation experiments in which three different molar ratios of recombinant Cactus was added to each Rel protein homodimer. Following immunoprecipitation each sample was separated by SDS-PAGE and visualized on immunoblots using an anti-His antibody. The first lane of the blot shows the molecular weight markers (M) in kilodaltons (kDa), while the second lane shows the control experiment, which lacked an NF-κB but contained all other co-immunoprecipitation components. Note that Cactus was not captured in the control experiment but was captured when recombinant Dif, Dorsal or Relish was present. The absence of Cactus with a 1∶ 1 molar ratio of Cactus∶ Relish suggests a lower affinity for this NF-κB than for Dif or Dorsal homodimers. The background band at ca. 50 kDa is due to the large amount of Protein A from the immunoprecipitation. (B and C) Ank proteins compete with Cactus for binding to recombinant Relish. Recombinant Cactus and Relish were incubated with increasing molar ratios of recombinant Ank-H4 (B) or Ank-N5 (C) followed by immunoprecipitation, SDS-PAGE separation and immunoblotting using an anti-His antibody. The molar ratio of Ank-H4 or Ank-N5 to Cactus is shown below each immunoblot.

Since the strongest binding interaction was between Cactus and Dif, we assessed whether traditional co-immunoprecipitation assays yielded similar trends by adding 3-fold molar excess of Cactus to Dif, Dorsal, and Relish followed by addition of an anti-thioredoxin antibody and protein A beads. Our results indicated that Cactus bound each Rel protein under these conditions, while dilution experiments suggested that Cactus bound Dif and Dorsal more strongly than Relish (Figure 3A). We then asked whether recombinant Ank-H4, Ank-N5, or Rel-49 could compete the binding of Cactus to different Rel homodimers. Rel-49, Ank-H4, and Ank-N5 could not compete the binding of Cactus to Dif or Dorsal under our reaction conditions when present at 200-fold molar excess (data not shown). In contrast, 15-fold molar excess of Ank-H4 reduced Cactus binding to Relish, and fully competed the binding of Cactus to Relish when present at 90-fold molar excess (Figure 3B). Despite exhibiting a lower binding affinity for Relish than Cactus or Rel-49 in our SPR assays, Ank-N5 also competed with Cactus for binding to Relish above 40-fold molar excess (Figure 3C). Rel-49 in contrast did not compete the binding of Cactus to Relish over the same range of concentrations (data not shown). Overall, these data indicated that Cactus bound Dif and Dorsal homodimers more strongly than Relish homodimers. They also indicated that Ank-H4 and –N5 bound each Rel homodimer with higher affinity than Rel-49, and bound homodimeric Relish more strongly than Cactus.

### MdBV Ank proteins inhibit processing of Relish

As previously noted, gel shift assays showed that Ank-H4 and –N5 inhibited binding of both Dif/Dorsal-containing NF-κBs to the to the κB site in the promoter of the *drosomysin* gene and Relish-containing NF-κBs to the κB site in the promoter of the *cecropinA1* gene [39]. These findings together with the preceding binding studies collectively suggest that Ank-H4 and –N5 disable Toll and Imd pathway signaling by binding to Dif/Dorsal- and Relish-containing NF-κBs. However, these data do not indicate which form of Relish Ank proteins interact with in vivo. Insect IκBs are thought to primarily bind NF-κBs in the cytoplasm of cells [1]. Studies from mammals, however, yield a more complicated picture with some IκB family members primarily localizing and binding Rel proteins in the cytoplasm (IκBε others binding NF-κBs in the cytoplasm and nucleus (IκBα and ß), and others still preferentially localizing to the nucleus and binding NF-κBs bound to DNA (Bcl-3) [45], [48], [59]. Thus, viral Ank proteins could bind compound Relish (Rel-110) in the cytoplasm, processed Relish (Rel-68) in the nucleus, or both.

**Figure 4:**
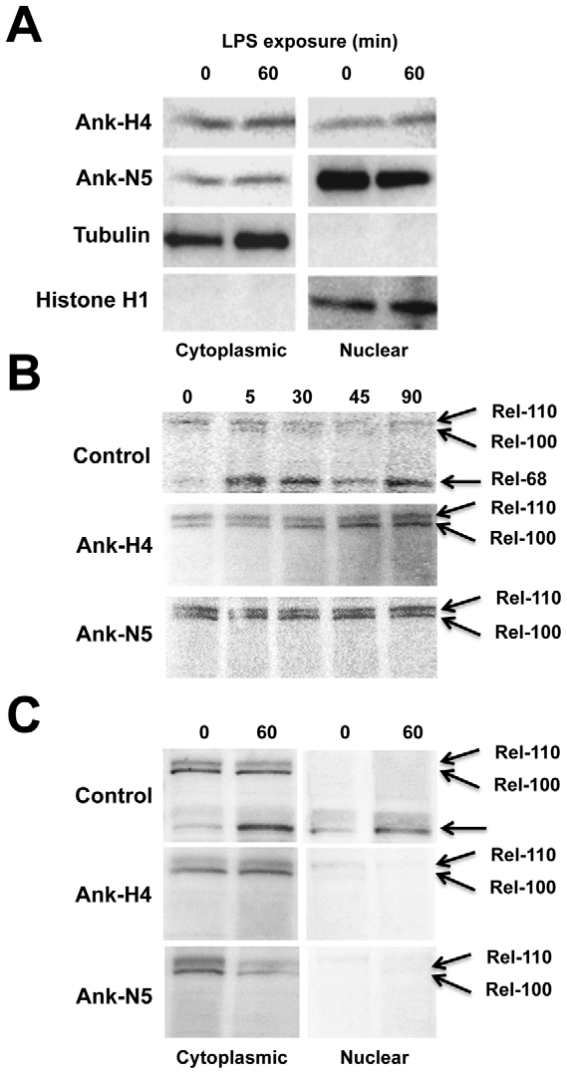
Ank proteins inhibit processing of compound Relish. (A) Immunoblots of cytoplasmic and nuclear extracts from mbn2 cells transfected with pIZT/Ank-H4 or pIZT/Ank-5. Cells were immune challenged with commercial LPS at 48 h post-transfection. Extracts were prepared 0 or 60 min post-challenge followed by SDS-PAGE and immunoblotting using an anti-His antibody to detect Ank-H4 and Ank-N5. Blots were also probed with an anti-ß Tubulin and anti-Histone H1 antibody. (B) Immunoblots of total cell extracts prepared from mbn2 cells transfected with pIZT/V5-His empty vector (Control), pIZT/Ank-H4 or pIZT/Ank-5. Cells were immune challenged 48 h post-transfection followed by preparation of extracts at 0, 5, 30, 45, and 60 min post-immune challenge. Blots were then probed with an anti-Rel-68 antibody, which recognizes compound (Rel-110/-100) and processed Relish (Rel-68). (C) Immunoblots of cytoplasmic and nuclear extracts from mbn2 cells transfected, immune challenged and processed as described in (B). The blot shows the presence of compound and processed Relish in samples prepared from control cells and cells expressing Ank-H4 or Ank-N5 at 0 and 60 min post-immune challenge.

We therefore transfected the expression constructs pIZT/Ank-H4, pIZT/Ank-N5, or pIZT (empty vector control) into *Drosophila* mbn-2 cells that have a functional Imd pathway. This pathway is also activated by commercial LPS which contains PGN [3], [20], [57]. We then prepared cytosolic and nuclear extracts from resting-state and LPS/PGN-challenged cells, followed by SDS-PAGE and immunblotting using an anti-V5 antibody to detect each Ank protein, and antibodies that detected the cytoplasmic protein ß Tubulin and nuclear protein Histone H1. Similar quantities of Ank-H4 were detected in the cytoplasmic and nuclear fractions of cells prior to (0 min) and 60 min after LPS/PGN challenge (Figure 4A). We also detected Ank-N5 in both fractions although its abundance was greater in the nuclear fraction (Figure 4A). The presence of these viral proteins in both fractions, however, was not due to sample preparation because we only detected ß Tubulin in our cytoplasmic fractions and Histone H1 in our nuclear fractions (Figure 4A).

Using total cell extracts and an anti-Rel-68 antibody [20], time course experiments showed that control and Ank protein-expressing cells contained full-length Relish (Rel-110) but little or no processed Relish (Rel-68) prior to LPS/PGN challenge (Figure 4B). We also detected a second 100 kDa band, which based on earlier studies corresponded to a full-length Relish variant (Rel-100) with a shorter N-terminus [20], [60]. Thereafter, we detected processing of Rel-110/-100 in control cells 5 min after exposure to LPS/PGN as evidenced by the appearance of Rel-68 (Figure 4B). In contrast, we detected no processing of Rel-110/-100 in cells expressing Ank-H4 or Ank-N5 over a 90 min assay period (Figure 4B). Examination of cytoplasmic and nuclear extracts from control cells at 0 and 60 min post-exposure to LPS/PGN confirmed that Rel-110/-100 remained in the cytoplasm, whereas Rel-68 was detected in both the cytoplasm and nucleus (Figure 4C). Rel-110/-100 also remained cytoplasmic in cells expressing Ank-H4 and –N5 (Figure 4C).

Combined with our SPR and co-immunoprecipitation data, these findings suggested that Ank proteins bind Rel-110/-100 in the cytoplasm, which in turn blocks formation and translocation of Rel-68 to the nucleus. An alternative explanation, however, could be that Ank proteins directly or indirectly inhibit the processing enzyme Dredd, which is a caspase-8 homolog [20], [21], [61], [62]. We compared Dredd activity in control cells and cells expressing Ank-H4 and –N5 using the substrate Ac-LETD-pNA. We readily detected caspase-8 activity but no differences were detected among treatments, which suggested that Ank proteins did not affect Relish processing activity (Figure S1).

### MdBV Ank proteins inhibit the inducible expression of multiple AMP genes

**Figure 5:**
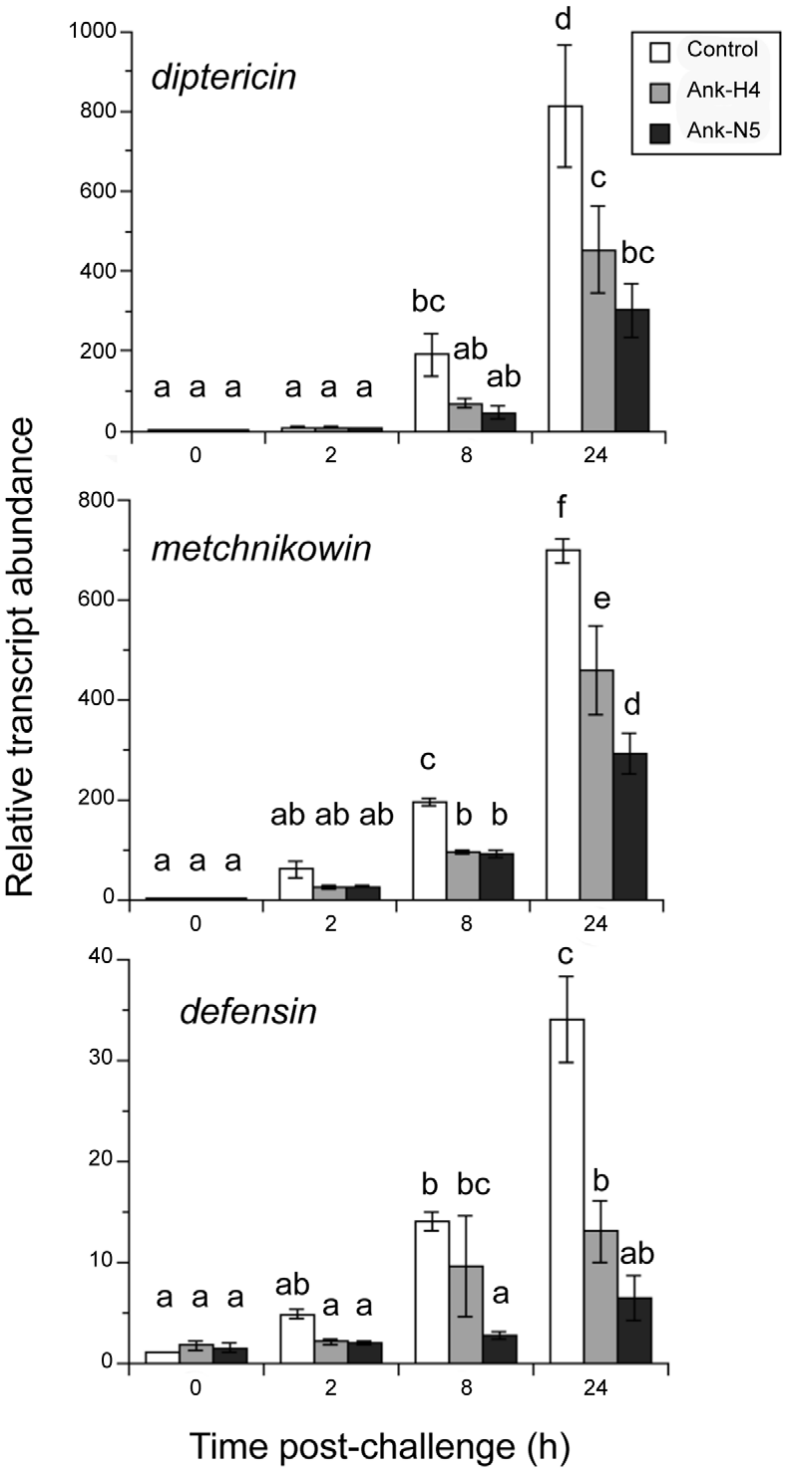
rqRT-PCR analysis of the AMP genes *diptericin*, *metchnikowin*, and *defensin* in mbn2 cells. Cells were transfected with pIZT/V5-His empty vector (Control), pIZT/Ank-H4 or pIZT/Ank-5 and then immune challenged with commercial LPS as described in Figure 4. Total RNA was then isolated from cells for each treatment at 0, 2, 8 and 24 h post-immune challenge. The 0 h Control sample was standardized to a value of 1. Transcript abundance for the other time points were then expressed relative to the 0 h Control. Each treatment and time point was measured three times using independently transfected samples. Error bars indicate ± SE. Different letters above a given bar indicates that transcript abundance significantly differs.

In addition to *cecropinA1*, other AMP genes activated by the Imd pathway and/or the Imd and Toll pathways include *diptericin*, *metchnikowin* and *defensin* [63]–[66]. To assess whether Ank proteins also reduced the expression of these read-out genes, we transfected mbn-2 cells with the aforementioned Ank expression constructs and then measured transcript abundance of each AMP gene after LPS/PGN challenge. As expected, transcript abundance of *diptericin* and *metchnikowin* increased greatly and *defensin* increased modestly in control cells transfected with the empty vector. However, transcript abundance of each AMP increased significantly less in cells expressing Ank proteins (Figure 5).

### MdBV infection inhibits the expression of AMP read-out genes in *Pseudoplusia includens*

As previously noted, our decision to use *Drosophila* Rel proteins as binding targets for MdBV Ank proteins was driven by a lack of functional data on NF-κB/IκB binding interactions in Lepidoptera generally and the natural host of *M. demolitor* (*P. includens*) in particular. We likewise used bacterial cell wall components (LPS/PGN) as an elicitor and AMP gene expression as read-outs in the preceding experiments, because the former is a known activator of the Imd pathway while the latter are well-characterized target genes. Of obvious interest though is whether these findings are relevant to natural parasitism. In the absence of MdBV infection, *P includens* mounts a potent immune response against *M. demolitor* that culminates in the encapsulation and death of wasp eggs 24–36 h after parasitism [34], [37], [67]. The pattern recognition receptors (PRRs) that recognize parasitoid wasps are unknown from any insect including *P. includens*, and it also remains unclear whether parasitism activates NF-κB signaling. Studies in the silkmoth *Bombyx mori*, however, indicate that bacterial elicitors induce the expression of AMP genes including *cecropin B1* and *lebocin 4*. Similar to *Drosophila*, the ortholog of Relish (BmRel2) from *B. mori* also binds κB sites in the promoters of these and other AMP genes [68]–[70]. We also previously identified *cecropin* and *lebocin* orthologs from *P. includens* and showed that immune challenge by heat-killed bacteria induces their expression [71].

**Figure 6:**
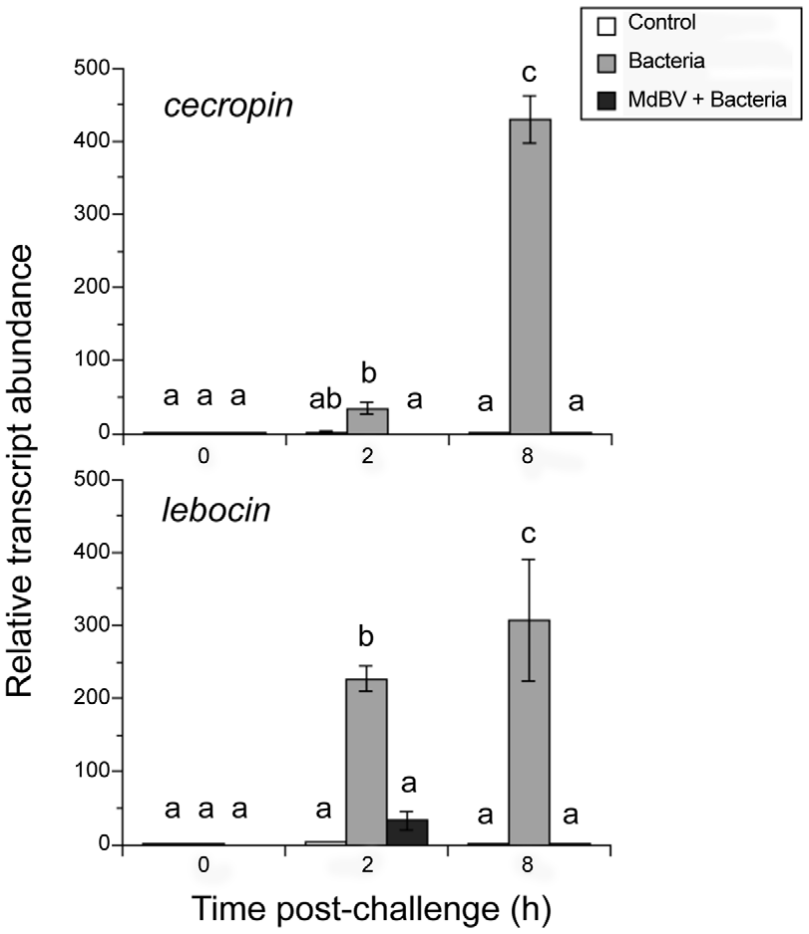
rqRT-PCR analysis of the AMP genes *cecropin* and *lebocin* in *P. includens* fat body. *P. includens* fifth instars were immune challenged with sterile PBS (Control) or heat-killed bacteria in sterile PBS (Bacteria), or were infected first with a physiological dose of MdBV followed 12 h later by injection of heat-killed bacteria (MdBV+Bacteria). Total RNA was then isolated from the fat body of each larva at 0, 8 and 24 h post-immune challenge with sterile PBS or bacteria. The 0 h Control sample was standardized to a value of 1. Transcript abundance for the other treatments and time points were then expressed relative to the 0 h Control. Each treatment and time point was measured three times using independently transfected samples. Error bars indicate ± SE. Different letters above a given bar indicates that transcript abundance significantly differs.

**Figure 7:**
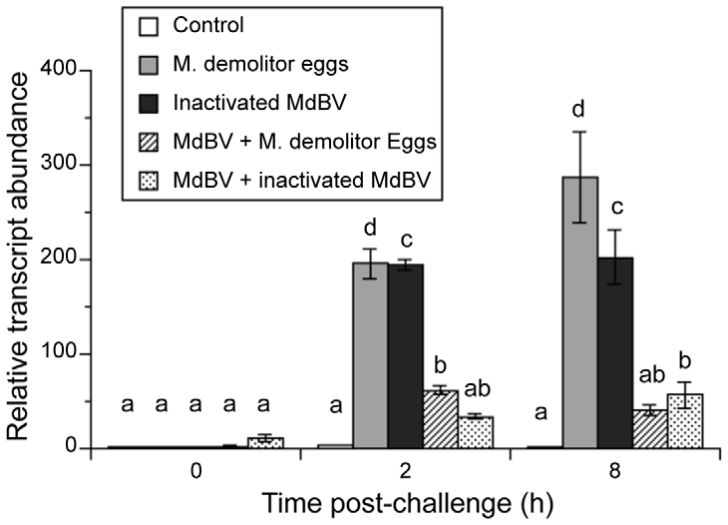
rqRT-PCR analysis of the AMP gene *lebocin* in *P. includens* fat body. *P. includens* fifth instars were either immune-challenged with sterile PBS (Control), *M. demolitor* eggs, or inactivated MdBV (Bacteria), or were infected first with a physiological dose of MdBV followed 12 h later by injection of *M. demolitor* eggs or inactivated MdBV. Samples were isolated and analyzed as described in Figure 6.

Taken together, these data suggest that *cecropin* and *lebocin* are potential read-out genes for activation of the Imd pathway in Lepidoptera. We therefore asked if MdBV infection disrupts *cecropin* and *lebocin* expression in *P. includens* after immune challenge by bacteria. Consistent with prior results, transcript abundance of both AMPs rapidly increased in the fat body of *P. includens* larvae following bacterial challenge relative to our wounding control (Figure 6). Bacterial challenge, however, did not induce the expression of these AMP genes if larvae had been infected 12 h earlier with a physiological dose of MdBV (Figure 6). We then assessed whether immune challenge by *M. demolitor* eggs and/or MdBV itself also induced the expression of these AMP genes. Similar to bacteria, wasp eggs and inactivated MdBV strongly stimulated the expression of *lebocin*, while pre-infection with MdBV near fully disabled this response (Figure 7). In contrast, wasp eggs and inactivated MdBV did not induce the expression of *cecropin*.

## Discussion

Vertebrate pathogens produce several virulence factors that target the innate immune system of hosts by mimicking proteins with essential signaling functions [23], [25], [26], [72], [73]. In some cases these mimics derive from host genes that the pathogen acquired and modified, while in others they share no significant homology with host proteins but through convergence have evolved similar structural features summarized by [26], [73]. Many invertebrate pathogens also subvert host immune defenses but in most cases the identity, function and origins of the virulence factors involved remain unknown [1], [24], [25], [74], [75].

NF-κB signaling is a key potential target for immune subversion in insects because the Toll and Imd pathways are widely conserved, respond to a diversity of infectious organisms, and regulate large numbers of immune genes [69], [76], [77]. Parasitoid wasps are among the most important mortality agents of insects, and more than 40,000 of these wasp species depend upon symbiotic PDVs for successful parasitism of hosts [78]. Strikingly, almost all PDV isolates studied to date encode *ank* genes [28], [31], while our own previous studies with MdBV indicated at least some *ank* genes function as IκB mimics [39].

Here we report the first kinetic measurements of insect IκB/NF-κB binding. Consistent with our own and earlier co-immunoprecipitation data [18], [19], [56], our SPR results indicate that Cactus binds the RHD and NLS domains of Dif and Dorsal with higher affinity than the same domains from Relish. Our SPR results also indicate that Ank-H4 binds Relish, Dif and Dorsal homodimers with similar affinity, while our competition experiments indicate that Ank-H4 and –N5 compete with endogenous IκBs to Relish. Among vertebrate family members, detailed kinetic studies have been conducted with recombinant IκBα and its NF-κB binding partners (p50/p65 or p65/p65) using SPR, isothermal titration calorimetry (ITC), and fluorescence polarization competition assays [40], [52], [79]. As we observe, these studies reveal very low dissociation rates for IκBα/NF-κB complexes, which are consistent with the long half-life these complexes exhibit in vivo [40]. The Kd values we determined are also broadly similar to those determined for IκBα/NF-κB homodimers (3–180 nM) but much lower Kd values have been determined for IκBα/NF-κB heterodimers (30–40 pM) than we detected. This suggests the possibility that binding interactions between IκBs and NF-κB homodimers may be weaker than those between IκBs and NF-κB heterodimers. However, the aforementioned vertebrate studies also indicate that the strength of IκB/NF-κB binding interactions in vitro is highly sensitive to salt concentration, temperature, and other testing conditions. Thus, the conditions we used in our binding studies could also be suboptimal, which could also explain why Ank-N5 competed Cactus binding to Relish in vivo but exhibited lower binding affinities for Relish than Cactus in vitro.

Our finding that Cactus most strongly binds Dif and Dorsal homodimers is fully consistent with the known role of these Rel proteins in regulating Toll signaling. In contrast, the significance of Cactus also binding Relish is less clear. Prior studies indicate that Relish processing is not be affected by RNAi knockdown of Cactus [80]. However, Relish does co-immunoprecipitate with Dif and Dorsal as a presumptive heterodimer, which form after processing [4], [55]. Given evidence from crystal structures of mammalian IκB/NF-κB complexes that IκBs contact both members of the dimer [15], it is thus possible that Cactus is functionally important in regulating Dif-Relish or Dorsal-Relish heterodimers. Another interesting feature of our binding data in regard to endogenous IκBs is that Rel-49 binds Dorsal, Dif and Relish homodimers, which parallel studies from mammals indicating that the IκB domain of p105 also binds its corresponding Rel domain after cleavage [50]. In contrast, it was not technically possible for us to assess whether Rel-49 also binds the Rel domain of Relish prior to processing. Thus, further studies will be required to understand the importance of Rel-49 in regulating the activity of compound versus processed Relish. Additional studies will also be needed to measure and understand the binding affinities of Cactus and Rel-49 for the Rel protein heterodimers that form in vivo [4], [15].

Our previous results [39] together with the binding data of this study collectively indicate that Ank proteins suppress both Toll and Imd pathway signaling by binding to Dif, Dorsal, and Relish-containing NF-κBs. Results of the current study further reveal that Ank proteins localize to both the cytoplasm and nuclei of mbn-2 cells, and disrupt Imd signaling by blocking processing of Rel-110/-100 in the cytoplasm rather than by interfering with Rel-68 in the nucleus. Notably, the inhibitory activities of vertebrate IκB family members also correlates more strongly with the efficiency that a given family member sequesters its target NF-κB in the cytoplasm than with its ability to inhibit binding of NF-κBs to DNA in the nucleus [59].

In *Drosophila* and mosquitoes, processing of compound Relish depends upon cleavage by the caspase-8 homolog Dredd while Dredd itself is negatively regulated by the FAF1 homolog Caspar [21], [62], [81]. We think it unlikely, however, that Ank proteins affect either Caspar or Dredd after immune challenge given we detected no reduction in Dredd/caspase 8 activity cells expressing Ank proteins. As noted above, these findings also raise important but unresolved questions about the role of the IκB domain in compound Relish in resting cells and whether Rel-49 functions as an IκB after processing. Although Rel-49 bound Rel protein homodimers in our SPR assays, the apparent ability of Ank proteins to bind and block processing of compound Relish in vivo suggests that the Rel-49 domain does not strongly interact with RHD and NLS of compound Relish either before or after immune challenge.

Our own unpublished transcriptome data identifies Rel gene homologs and most other components of the Toll and Imd pathways in *P. includens*. However, in the absence of any background studies on IκB/NF-κB interactions at the protein level, we currently are not able to directly determine whether MdBV Ank proteins bind to and disable NF-κBs in this natural host of *M. demolitor*. However, prior studies do indicate that *ank-H4* and *–N5* are rapidly and persistently expressed in the fat body and hemocytes of *P. includens* after MdBV infection [33], while results of the current study show that MdBV infection inhibits the expression of two AMP genes. Studies from *B. mori* suggest these AMP genes are likely regulated by the Imd pathway, although in *P. includens* we recognize the possibility they could also be regulated fully or in part by Toll signaling. We also show that immune challenge with *M. demolitor* eggs or inactivated MdBV strongly induces the expression of one of these AMP genes (*lebocin*) and that MdBV infection blocks this response. These results are fully consistent with our results in *Drosophila* cells, which show that Ank-H4 and –N5 disable both Imd and Toll signaling [39]. Results of the current study also suggest parasitism activates NF-κB signaling in the natural host but MdBV subverts this response.

Other viruses and parasitoids are known to also activate NF-κB signaling [8], [9], [11], [12], [81], [82], but no studies to our knowledge indicate that AMPs are important effector molecules in defense against these entities. However, NF-κBs regulate many other genes in response to infection whose function remains unknown [11], [76], [83], [84]. Thus, while MdBV disables expression of AMP genes, it is likely that other genes with roles in anti-parasitoid or anti–viral defense underlie the benefits to *M. demolitor* of subverting NF-κB signaling. Given that MdBV encodes other virulence genes that disable hemocyte function and the phenoloxidase (PO) cascade [35]–[38], it is also likely that *ank* genes interact with other MdBV gene products to disable both cellular and humoral defense responses of hosts.

The Imd and Toll pathways are thought to also play important roles in defending insects against opportunistic microbes, which most commonly infect insects by oral ingestion [1]. Thus, a possible cost to suppressing NF-κB signaling could be that it renders hosts and a developing parasitoid more susceptible to infection by other organisms. However, infection of hosts by PDVs also induces profound alterations in behavior including a near complete cessation of feeding, which likely reduces the risks of infection by another pathogen before the wasp's progeny complete their development [28].

Studies of two other PDVs implicate Ank proteins in inhibition of NF-κB dependent transcriptional activity [85], [86], while comparative data show that some Ank protein family members localize to the cytoplasm of insect cells, others localize to nuclei, and others still localize to both [85]–[87]. PDVs like MdBV belong to the genus *Bracovirus*, which evolved more than 100 million years ago from another taxon of viruses that infect insects called nudiviruses [30], [31], [88], [89]. Comparative genomic data further indicate the largest and most conserved genes encoded by bracoviruses are the *ank* and *ptp* gene families. The absence of any genes with significant homology to *ank* genes among known nudiviruses suggests this gene family originated from a eukaryote where it potentially functioned as an IκB. However, the ancient origins of the *ank* family together with rapid rates of evolution make it unclear whether this eukaryote was a wasp, an insect host, or another organism that predates the evolution of the Hymenoptera [31], [90], [91].

## Materials and Methods

### Ethics statement

All studies were approved by the Biological Safety and Animal Care and Use Committee of the University of Georgia and were performed in compliance with relevant institutional policies, National Institutes of Health regulations, Association for the Accreditation of Laboratory Animal care guidelines, and local, state, and federal laws.

### Insects, MdBV isolation, and collection of *M. demolitor* eggs

*M. demolitor* and *P. includens* were reared as previously described [92]. MdBV and MdBV genomic DNA were isolated from adult female *M. demolitor* as outlined by [93]. MdBV was transcriptionally inactivated by UV light treatment [94], while *M. demolitor* eggs were collected aseptically from female wasps in sterile phosphate-buffered saline (PBS) [37].

### Cloning and recombinant protein expression

For bacterial expression of Dif and Dorsal, ORFs containing the RHD, nuclear localization signal (NLS), and 20 amino acids downstream of the NLS were polymerase chain reaction (PCR)-amplified using gene specific primers with sequence extensions for cloning into pET-LIC vectors (Table S1). The plasmids pSHhis-Dif and pSHhis-Dorsal respectively served as templates [4]. Full length Relish and Rel-49 were similarly amplified using gene specific primers and the plasmid pSHflag-Relish as template [4]. Each of the aforementioned plasmids was obtained from T. Ip (University of Massachusetts). The ARD of Cactus was amplified using specific primers and a full-length cDNA clone of Cactus (LD10168) from the *Drosophila* Genomics Resource Center as template, while Ank-H4 and Ank-N5 were amplified using specific primers and MdBV genomic DNA as template (Table S1). Briefly, 1 ng of template, 250 nM of each primer and 1.2 Units of KOD HiFi DNA Polymerase (Novagen) were combined in a 50 µl volume and amplified using the following conditions: 25 cycles of 98°C for 15 sec, 61°C for 2 sec, and 72°C for 20 sec. Rel products were then cloned into either pET32-EK-LIC, which encodes an N-terminal Thioredoxin and 6× histidine (His) affinity tag or pET51-EK-LIC, which encodes an N-terminal StrepTagII affinity tag. The IκB domain of Relish (Rel-49) was cloned without a stop codon into pET51-EK-LIC resulting in a C-terminal His tag. Cactus, Ank-H4, and Ank-N5 in contrast were cloned into pET-30-EK-LIC, which encodes an N-terminal His tag. Each construct was confirmed by DNA sequencing, and then expressed by transforming into *Escherichia coli* strains BL21 (DE3) cells. Transformed *E. coli* were grown in 2 L Luria Broth containing 100 µg/ml ampicillin (pET32 and pET51) or 50 µg/ml kanamycin (pET30) at 37°C with shaking at 275 rpm until the A_600_ reached 0.8–1.0. The cultures were cooled to room temperature and then induced with 0.1 mM isopropyl-β-d-thiogalactopyranoside (IPTG) for an additional 4–24 h at 20°C. Bacterial cells were harvested by centrifugation at 5000× *g* for 10 min and used immediately or stored at −80°C.

Bacterial pellets from 0.8 L cultures were resuspended in 40 ml of lysis buffer (50 mM Tris-HCl pH 8.0, 300 mM NaCl, 10 mM imidazole). After addition of lysozme (1 mg/ml) in 50 mM Tris-HCl (pH 8.0), cells were incubated on ice for 1 h followed by sonication with six, 10 sec bursts at 200 W using a Branson 450 Sonifier. For the constructs containing His affinity tags, the soluble recombinant proteins were purified from the clarified supernatant by incubating with 2 ml Ni-NTA Supreflow (Qiagen) agarose beads for at least 2 h and mixing by tumbling end over end at 4°C. The beads were then pelleted by centrifugation at 500× *g* for 5 min, and the supernatant (flow through) was removed. The beads were then resuspended with an equal volume of Buffer A (20 mM Tris–HCl, pH 8.5, 0.5 M KCl, 5 mM β-mercaptoethanol, 10% glycerol, 20 mM imidazole) at room temperature and quantitatively transferred to a 15 ml column at room temperature. The column was then packed at 1.5 ml/min with buffer A until the bed volume was constant, then washed with 10 volumes of buffer A at 1 ml/min, followed by two volumes of buffer B (buffer A, containing 1 M KCl and no imidazole). The column was then washed with two more volumes of buffer A, and the protein was eluted with buffer C (buffer A with 100 mM imidazole). Fractions (1.0 ml) were analyzed by SDS–PAGE and immunoblotting using an anti-His monoclonal antibody (Sigma or Qiagen) and stored at 4 or −80°C. When necessary, remaining contaminating proteins were removed by gel filtration using a Superdex75 column (Amersham). Proteins with an N-terminal StrepTagII were isolated using Streptactin agarose (Novagen). Briefly, bacterial lysates were prepared as described above and applied to a 2 ml packed and equilibrated Streptactin column at 0.5 ml/min at 4°C. The column was washed with 40 ml of wash buffer (50 mM Tris, pH 8, 150 mM NaCl, 5 mM 2-mercaptoethanol) at 0.5 ml/min at 4°C. Proteins was eluted with 10 ml of elution buffer (wash buffer with 2.5 mM desthiobiotin) and stored at 4° or −80°C. Proteins were desalted with PD-10 columns (Amersham) and washed into appropriate buffers using spin filtration. Protein concentrations were determined using the Pierce Coomassie Plus Bradford assay.

### Surface Plasmon Resonance

Biosensor experiments were run on a Biacore 3000 instrument (GE Healthcare) at room temperature. Recombinant ligands (usually IκBs) were immobilized on research grade CM5 sensor chips by amine coupling as follows. The carboxymethyl surface of the chip was activated for 8 min at 5 µl/min with a 1∶1 mixture of 0.4 M N-ethyl-N′-(3-dimethylaminopropyl) carbodiimide (EDC) and 0.1 M N-hydroxysuccinimide (NHS). Recombinant IκBs diluted to 10 µg/ml in 10 mM sodium acetate, pH 4.5 were injected using quickinject in 5 sec pulses until a surface density of 5500 response units was achieved. Excess activated succinyl groups were then blocked by injecting 1 M ethanolamine, pH 8.5 for 8 min at 10 µl/min.

Kinetic titration experiments were performed by serially diluting recombinant analytes (usually NF-κBs) in running buffer (10 mM HEPES, pH 7.4, 150 mM NaCl, 3.4 mM EDTA, 0.005% (v/v) plus surfactant P20), and sequentially injecting doubling concentrations for 60 or 90 sec, allowing 120 sec dissociation after each injection. Injections were made across both the ligand bound cell and a reference cell, in which the surface had been activated with EDC: NHS, and then immediately deactivated with ethanolamine. Sensorgrams were recorded by automatic subtraction of the blank reference cell from the experimental cell to remove non-specific binding affects and to correct for drift. Typically, five injections of 160 nM through 2.56 µM NF-κB were measured. The response profiles were fit to the kinetic titration model (provided by Biacore) assuming simple 1∶1 Langmuir binding to generate kinetic and thermodynamic binding constants. The high surface densities used were necessary to produce clean responses above the noise of the machine. To determine whether mass transport effects significantly influenced results, the Dif-Cactus interaction was analyzed using a surface density of 15,000 response units (RU). The constants measured were within the standard error of the experiments using a ligand surface density of 5000 RU. Therefore, mass transport effects were deemed negligible.

### Co-immunoprecipitation experiments

All incubations were performed at room temperature on a rotator. Rel protein homodimers were diluted into binding buffer (50 mM Tris, pH 8, 150 mM NaCl, 0.1% BSA, 0.1% Triton X-100) to a concentration of 4.8 nM, and incubated with varying concentrations of competing IκBs for 1 h. Cactus was initially added to 14.3 nM, a 3 fold molar excess of the Rel protein, and incubated for 1 h. Rabbit anti-thioredoxin antibody (0.5 µl, Sigma T 0803) was then added and incubated for 1 h. Protein A beads (BioRad Affigel) were equilibrated in binding buffer and 20 µl of equilibrated, packed beads were added to the reactions and incubated for 1 h. The beads were pelleted by centrifugation at 1000× *g*, the supernatant was discarded, and the beads were washed 3× with binding buffer. The beads were then washed a fourth time with binding buffer with no BSA or Triton X-100 and the supernatant discarded followed by suspension in 50 µl of 1.5× SDS-sample buffer plus 2-mercaptoethanol and boiled for 5 min. The resulting supernatants were then subjected to SDS-PAGE and immunoblot analysis as described below.

### Transfection of *Drosophila* mbn-2 cells, cell extracts, and RNA isolation

The coding sequences for *ank-H4* and *ank-N5* were previously cloned into the expression vector pIZT/V5-His (Invitrogen), which uses the OpIE2 promoter from *Orgyia pseudotsugata* baculovirus for constitutive expression of the gene of interest and incorporates a C-terminal V5 epitope tag [39]. *Drosophila* mbn-2 cells were maintained in Schneider's medium (Sigma) supplemented with 10% fetal bovine serum (Atlanta Biologicals) [20]. Mbn-2 cells were transfected by adding cells to 6 well culture plates (Corning) (1×10^6^ cells per well in 1 ml of complete medium). Twenty-four h later, 2 µg of each construct (pIZT/Ank-H4, pIZT/Ank-N5, or pIZT/V5-His (empty vector)) was diluted into 1 ml of Schneider's medium without serum followed by addition of 16 µl of Cellfectin (Invitrogen). After a 20 min incubation period, complete medium was removed from the cells in each well and the transfection medium was added. The transfection medium was then removed after 6 h and replaced with 1 ml of complete medium. Cells were immune challenged 48 h post-transfection with 10 µg/ml of commercial lipopolysaccharide (LPS) that contained peptidoglycan (PGN) (Sigma) for 2–24 h. Following collection and centrifugation, cell pellets were washed 3× in PBS (pH 7.2). Whole cell lysates were prepared by resuspending cell pellets in lysis extraction buffer (20 mM HEPES, pH 7.5, 100 mM KCl, 0.05% Triton X-100, 2.5 mM EDTA, 5 mM DTT, 5% glycerol, and protease plus phosphatase inhibitor cocktail (Roche). Cytoplasmic and nuclear extracts were prepared using NE-PER Nuclear and Cytoplasmic Isolation Kit (Pierce) plus protease and phosphatase inhibitor cocktail. Protein concentrations were determined by Bradford assay. Total RNA was isolated from mbn-2 cells using the Hi-Pure RNA extraction kit (Roche) and quantified using a Nanodrop spectrophotometer (Thermo Scientific).

### SDS-PAGE and immunoblotting

For analysis of recombinant proteins, samples were electrophoresed on 1 mm PageR precast minigels (Lonza) and transferred to PVDF (Immobilon) by tank transfer. The membranes were blocked for 1 h in 5% dry milk in TPBS (0.1% Tween 20), followed by detection using a mouse anti-His monoclonal antibody (1: 2000) (Qiagen) and a goat anti-mouse horseradish peroxidase-conjugated secondary antibody (Jackson Laboratory) (1∶20,000). Bands were visualized using 3, 3-diaminobenzidine. For analysis of cell extract proteins, samples (20 µg of protein per lane) from mbn-2 cells were electrophoresed on precast 4–20% gradient gels (Lonza) followed by transfter to PVDF membranes and blocking as described above. Ank-H4 and -N5 were detected using a murine anti-V5 antibody (Invitrogen) (1: 10,000) and a goat anti-mouse horseradish peroxidase-conjugated secondary antibody (Jackson Laboratory) (1∶20,000). β-tubulin and Histone H1 were detected using a goat anti-β tubulin polyclonal antibody (Abcam) (1∶5000) or mouse anti-Histone H1 antibody (Santa Cruz) (1∶1000) followed by incubation with a goat or mouse anti-rabbit horseradish peroxidase-conjugated secondary antibody (1∶10,000 or 1∶5000). Relish was detected using a rabbit anti-Rel-68 antibody (S. Stoven, University of Umea) and anti-rabbit horseradish peroxidase-conjugated secondary antibody (1∶10,000. Bands were visualized by chemiluminescence using the ECL Advance Western blotting detection kit (Amersham Biosciences) and a bio-imaging system (Syngene).

### Relative quantitative real-time PCR (rqRT-PCR)

First-strand cDNA was synthesized from mbn-2 cell total RNA using random hexamers and Superscript III (Invitrogen) [33]. rqRT-PCR reactions were run using a Rotor-Gene 3000 Cycler (Corbett) with 10 µl reaction volumes containing 1 µl of cDNA, 5 µl of iQ SYBR Green Supermix (Bio-Rad) and 250 nM of forward and reverse primers specific for the AMP genes *diptericin*, *metchnikowin*, and *defensin*, or the *Drosophila* 18 s ribosomal gene (Table S1). Cycling conditions were: initial denaturation at 94°C for 3 min, followed by 45 cycles with denaturation at 94^•^C for 10 sec, annealing at 50/55^•^C for 15 sec, and extension at 72°C for 20 sec. Data were acquired during the extension step, and analyzed with the Rotor-Gene application software. For every amplicon, reactions were carried out in quadruplicate, from which mean threshold cycle (C_T_) values plus standard deviations were calculated. All data were normalized to internal 18 s rRNA levels from the same sample. To compare transcript abundance for a given gene among treatments, we calibrated each ΔC_T_ value against 0 h control, generating a ΔΔC_T_ value, followed by transformation using the expression 2^−ΔΔCT^ to obtain relative transcript abundance values (RA) [95]. In some cases these data were non-normally distributed. We therefore used a natural log transformation of each RA followed by ANOVA and pairwise t-tests to assess differences among treatments [33].

### Caspase-8 assays

Dredd activity in mbn-2 cells transfected with pIZT/Ank-H4, pIZT/Ank-N5, or empty vector was assessed using a commercially available caspase-8 assay (Caspase-Glo) and the luminogenic substrate Ac-LETD-pNA (Promega) according to the manufacturer's protocol. All assays were performed in duplicate using independent samples and a BioTek Synergy 4 plate reader. Relative luminescence units (RLU) were determined 10 min after addition of substrate with the resulting data thereafter analyzed by ANOVA.

### Infection of *P. includens* larvae and rqRT-PCR assays

*P. includens* fifth instars (day 2) were immune challenged by injecting larvae with heat killed *E. coli* (1×10^6^ cell in 1 µl of PBS), a physiological dose of inactivated MdBV (1×10^9^ virions ( = 0.1 wasp equivalents) in 1 µl PBS [93], or 3–5 *M. demolitor* eggs in PBS using a glass needle mounted on a micromanipulator. Larvae injected with sterile PBS alone served as a wounding control. Other larvae were first injected with 0.1 wasp equivalents of viable MdBV followed 12 h later by immune-challenge using the above elicitors. Fat body was dissected from individual larvae in sterile PBS either before challenge with each elictor (0 h) or 2 and 8 h after. Isolation of total RNA, first-strand cDNA synthesis, and rqRT-PCR reactions were then run using primers specific for the *P. includens cecropin*, *lebocin* or 18 s ribosomal RNA gene (Table S1) as described above.

## Supporting Information

Figure S1 **IETDase activity in mbn2 cell extracts.** Cells were transfected with pIZT/V5-His empty vector (Control), pIZT/Ank-H4 or pIZT/Ank-5 and then immune challenged with commercial LPS 48 h post-transfection. Extracts were then prepared followed by addition of substrate and measurement of relative luminescence units (RLU) after 10 min at 25°C. Each treatment was performed in duplicate using independent samples. No differences in activity were detected among treatments (F_5,17_ = 1.50; P = 0.3).
(TIF)

Table S1 **Primers used for construction of expression constructs, and in rqRT-PCR assays.**
(DOCX)
