# Supplementary figures and images for: Polydnavirus Ank Proteins Bind NF-κB Homodimers and Inhibit Processing of Relish
Source: PLoS Pathog. 2012 May 24;8(5):e1002722. doi: 10.1371/journal.ppat.1002722 (PMC3359993; doi:10.1371/journal.ppat.1002722)

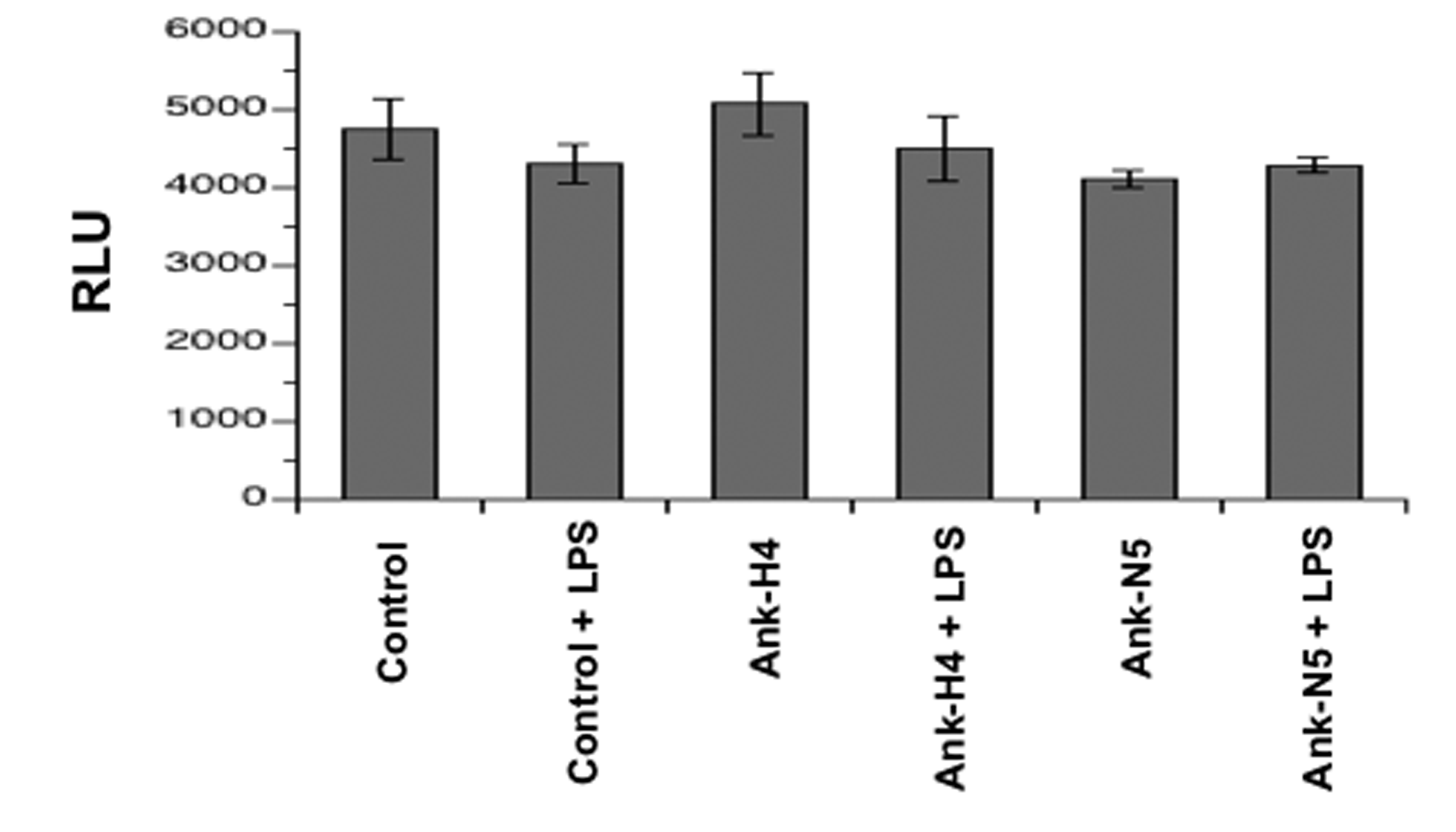

Supplement: Figure S1 — IETDase activity in mbn2 cell extracts. Cells were transfected with pIZT/V5-His empty vector (Control), pIZT/Ank-H4 or pIZT/Ank-5 and then immune challenged with commercial LPS 48 h post-transfection. Extracts were then prepared followed by addition of substrate and measurement of relative luminescence units (RLU) after 10 min at 25°C. Each treatment was performed in duplicate using independent samples. No differences in activity were detected among treatments (F5,17 = 1.50; P = 0.3). (TIF) [file ppat.1002722.s001.tif]
