# Supplementary material for: Polydnavirus Ank Proteins Bind NF-κB Homodimers and Inhibit Processing of Relish
Source: PLoS Pathog. 2012 May 24;8(5):e1002722. doi: 10.1371/journal.ppat.1002722 (PMC3359993; doi:10.1371/journal.ppat.1002722)
Supplement: Table S1 — Primers used for construction of expression constructs, and in rqRT-PCR assays. (DOCX) [file ppat.1002722.s002.docx]

**Table S1. Primers used for construction of expression constructs, and in rqRT-PCR assays.**

| Primers used for expression constructs | Forward (5’- 3’) | Reverse (5’- 3’) |
| --- | --- | --- |
| Rel constructs |  |  |
| *dif* | GACGACGAAAGATTCCCCACCTGCGTATCGTGGAGGAG | GAGGAGAAGCCCGGTTCATGGATGTAAATTGGTGCTCAT |
| *dorsal* | GAGGAGAAGCCCGGTTCATGGATGTAAATTGGTGCTCAT | GAGAGAAGCCGGTTCAATTCTGCAATTGCTGTTTCTG |
| *relish* | GACGACGACAAGATGCCGCAGCTGCGGATCGTTA | GAGAGAAGCCCGGTTCAATCCAGCGAGTTATTAGAGCTTTC |
| IκB constructs |  |  |
| *cactus* | GACGACGACAAGATCTGGGAGCAGTTCTATCAACAAAAC | GAGGAGAGCCCGGTCAAAAGCGATCGTACATCTTCGTATC |
| *rel-49* | GAGACGACAAGATTGGTCATAACCGGGCGGAAGTGCC | GAGGAGAAGCCCGGTAATCCAGTTGGGTTAACCAGTAG |
| Ank constructs |  |  |
| *ank-H4* | GACGACGACAAGATGGTGCGATACTA | GAGGAGAAGCCCGGTTTAGGATACATTTTTTTC |
| *ank-N5* | GACGACGACAAGATGGAGCGTGCAGATAATTC | GAGGAGAAGCCCGGTCTACTTGAACAATTGCATCATATAAG |
| Primers used in rqRT-PCR  assays with cDNA from  *Drosophila* mbn2 cells |  |  |
| *Dm diptericin* | ATTGCCGTCGCCTTACTT | TCGGAAATCTGTAGGTGTAGGT |
| *Dm metchnikowin* | CTTAATCTTGGAGCGATTTTTC | AATAAATTGGACCCGGTCTTG |
| *Dm defensin* | CGCAGACGGCCTTGTC | AAGTTCTTCGTTCTCGTGG |
| *Dm 18s* | AACGGCTACCACATCTAAGGAAGG | CCGGCCCACAATAACACTCG |
| Primers used in rqRT-PCR  assays with cDNA from  *P. includens* fat body |  |  |
| *Pi cecropin* | GCAAGCAGCCGCCATCA | TACACATCCAATACTCAACATCAA |
| *Pi lebocin* | TGTGTTGTCGGCGTTCTT | CTCTCCCCATGCTCAATCTC |
| *Pi 18s* | CAGTGATGGGATGAGTGCTTTTATTAGAT | AGGCCCTCCGTCGATTGGTTTT |
